# The protective impact of betaine on the tissue structure and renal function in isoproterenol‐induced myocardial infarction in rat

**DOI:** 10.1002/mgg3.579

**Published:** 2019-02-27

**Authors:** Mohammad Maram Ghartavol, Shiva Gholizadeh‐Ghaleh Aziz, Ghader Babaei, Gholam Hossein Farjah, Mohammad Hassan Khadem Ansari

**Affiliations:** ^1^ Department of Clinical Biochemistry Urmia University of Medical Sciences Urmia Iran; ^2^ Department of Anatomy Urmia University of Medical Sciences Urmia Iran

**Keywords:** betaine, creatinine, isoprenaline, kidney, myocardial infarction, NOS, TNF‐α

## Abstract

**Background:**

Myocardial infarction is one of the most common life threatening diseases that may lead to renal disorders via oxidative stress and inflammation. Betaine is a safe and well‐tolerated compound exhibiting beneficial antioxidant and anti‐inflammatory properties. Previous studies have demonstrated protective effects of betaine against myocardial infarction and renal injury. This study aimed to investigate the protective effect of betaine on tissue structure and renal function after isoprenaline‐induced myocardial infarction in rats.

**Methods:**

Fifty Wistar strain male albino rats, weighing 200 ± 10, were selected for the study. The animals were housed individually under standard environmental conditions (Light–dark cycle, temperature and constant humidity) for 1 week. After acclimatization, they were randomly divided into five groups. G1, G2, and G3 groups received betaine at doses of 50, 150, and 250 mg/kg body weight/day via gavage for a period of 60 days. After 60 days, isoprenaline is injected subcutaneously (200 mg/kg body weight). In the isoprenaline group (G4), the rats were injected with isoprenaline (200 mg/kg body weight) and the control group (G5) received a standard diet (Without isoprenaline). Then, isoproterenol solution was used for induction of myocardial infarction. At the end, the expression of nitric oxide synthase (iNOS) protein was detected using immunohistochemical analysis and kidney tissues were assessed via histopathological analysis. In addition, serum level of TNF‐α and creatinine level were measured via ELISA test and colorimetric methods, respectively.

**Results:**

The results of our study indicate that isoproterenol‐induced renal histopathological injury without changing creatinine level. Betaine has protective effects against renal injuries induced by isoprenaline and the expression of nitric oxide synthase (nNOS) protein showed no significant difference in all groups. Further, betaine reduced TNF‐α level significantly.

**Conclusion:**

According to our results, betaine has protective effects on isoprenaline‐induced renal failure via a decrease in TNF‐α level and nitric oxide synthase.

## 1 INTRODUCTION

Noncommunicable diseases ranked among the top 15 diseases in 1990, but estimates indicate that they will rank among the first six diseases in 2020 (Lim et al., 2012). Myocardial infarction is a process that is caused by stoppage or reduced coronary arteries following the blockage of one of the branches of the coronary arteries and sudden stoppage of blood flow and lack of oxygen to the heart muscle (Thygesen, Alpert, & White, 2007). Thirty‐six percent of deaths in Iran is caused by cardiovascular diseases where myocardial infarction being the most important cause (Nizal Sarrafzadegan, Sadeghi, Shahram Oveisgharan, & Marshall, 2013). Studies have shown that, in addition to the heart, myocardial infarction afflicts other organs including the brain, liver, and kidneys (MOROOKA et al., 1992). Damage to both the heart and kidneys is called cardiorenal syndrome. Generally, the heart and kidneys have complex interactions physiologically and endocrinologically; in other words, dysfunction of the heart affects kidneys and vice versa. Based on which organ is damaged first, the result is divided into five acute or chronic conditions: (1) acute or type one in which acute function of the heart causes kidney dysfunction; (2) chronic or type two in which chronic function of the heart causes kidney dysfunction; (3) acute function of the kidney causes heart dysfunction; (4) chronic function of the kidney causes heart dysfunction; (5) in this type, systemic problems lead to dysfunction of both kidneys and heart. Next to myocardial infarction, kidney damages and dysfunction is type 1 cardiorenal syndrome (Ronco, Haapio, House, Anavekar, & Bellomo, 2008). In term of pathology, oxidative stress and inflammation are the major causes of myocardial infarction. The severity of damages caused by myocardial infarction is directly correlated with these two variables. Betaine or Trimethylglycine is highly endurable for the body and has antioxidant and anti‐inflammatory properties. Its main role is methyl metabolism in the synthesis of compounds such as phospholipids, adrenal hormones and DNA/RNA and other mechanisms (Obeid, 2013). Previous studies have proved the protective role of Betaine in heart tissue following induced‐myocardial infarction by isoprenaline. Creatinine is the byproduct of muscle metabolism and is excreted by kidneys, and the level of this factor in the serum is considered a good index for kidney function (Samra & Abcar, 2012). Tumor necrosis factor alpha (TNF‐α) is an inflammatory cytokine; it plays a vital role in the pathogenesis of cardiovascular and renal function (Fahim, Halim, & Kamel, 2004; Feng, Lu, Jones, Shen, & Arnold, 2001). Moreover, the effects of nitric oxide synthase cannot be ignored in terms of oxidative damage. Since reduced inflammation and oxidative stress may prevent kidney damage after infarction, the aim of the present study was to investigate the protective effects of Betaine on kidney injury caused by induced‐myocardial infarction by isoprenaline, creatinine level, TNF‐α serum level, and tissue nitric oxide synthase enzyme.

## 2 MATERIALS AND METHODS

Fifty Wistar strain male albino rats were selected for the study. The animals were provided from the animal house of Urmia University of Medical Sciences and were housed individually under standard environmental conditions (temperature‐controlled room [22–25°C] and 12 hr light–dark photo cycle) and received adequate food and water. After acclimatization, the experimental animals were divided into five groups, comprising 10 rats each (control group, MI group and the group treated with betaine at doses of 50, 150, and 250 mg/kg body weight). Three groups of 10 rats, as an intervention group were examined with betaine at doses of 50, 150, and 250 mg/kg body weight for a period of 60 days. After 60 days, isoprenaline was injected subcutaneously (200 mg/kg body weight). Rats in the normal control group received a standard diet and in the isoprenaline group, the rats were injected with isoprenaline (200 mg/kg) for 24 hr without pretreatment. Then noninvasive tail‐cuff blood pressure (BP) measurement (PowerLab system) showed decrease of systolic blood pressure of mice (mean ± *SEM*) from 100 ± 6 to 53 ± 7 mm Hg. Serum and tissue samples were isolated and stored at −80°C.

### 2.1 Histopathological and immunohistological assays

The tissues needed for the study were prepared according to the guidelines consisting of several steps: fixation, dehydration with various concentrations of alcohol, clarification, embedding, sectioning, and staining of hematoxylin and eosin. Immunohistochemical tests were used to identify the level of nitric oxide synthases (NOS). After the removal of tissues, the fixation was first carried out using formalin (10%). Then, the tissues were dehydrated with various concentrations and the ethanol was gradually replaced with xylene. Incubation was performed with an iNOS primary antibody (ABCAM) and a horseradish peroxidase‐conjugated secondary antibody. Diaminobenzidine substrates were identified to detect the target protein.

### 2.2 Measurement of serum TNF‐α levels and serum creatinine

The serum creatinine level was assessed using a colorimetric method with a wavelength of 500 nm at 37°C according to the kits performance characteristics from Pars Azmoon Company, Iran. First, proteins were removed based on colorimetric Jaffe method. According to this method, Picric acid in an alkaline medium reacts with creatinine to form an orange colored complex with the alkaline picrate. Intensity of the color formed is directly proportional to the amount of creatinine present in the sample. Elisa kit (cat no: E0082hu) was used to measure the TNF‐α level. Based on the Kite protocol instructions, after preparing the plates, 100 μl of buffer solution was added to the sample and then the solution was kept at room temperature for 2 hr. The sample was aspirated and washed in two steps and then 200 μl of the substrate solution was added to each well and was incubated at room temperature for 2 hr. Then, 100 μl of antibody solution was added to each well and stored at room temperature for 1 hr. After repeating the aspiration/wash in two steps, 200 μl of the substrate solution was added to each well and incubated at room temperature for 20 min. Finally, 500 µl of stop solution was added to each well. The optical density of each plate was determined using an ELISA reader set at 450 nm wave length (Donnahoo et al., 1999).

### 2.3 Statistical analysis

The data were first examined using S‐K (kolmogrov–smirnov) method to determine the distribution normality. Then the results were analyzed using R software. In addition, if the data were normally distributed, one‐way ANOVA method was used to compare the different groups. Kruskal–Wallis method, which is roughly equivalent to a parametric one‐way ANOVA, was used if data were not normally distributed. In each test, the data are expressed as the mean ± *SEM* and a *p* < 0.05 was considered as statistically significant.

## 3 RESULTS

### 3.1 Assessment of tissue damage by hematoxylin and eosin staining

**Figure 1:**
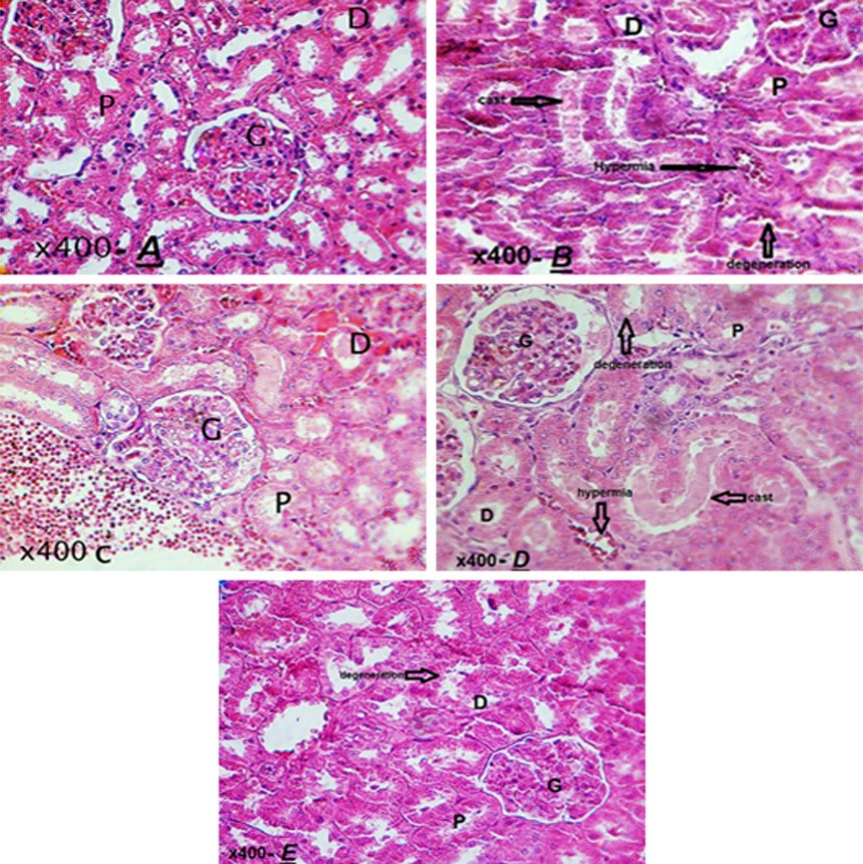
(A–E) Histopathologic section of hematoxylin and eosin‐stained kidney in the myocardial infarction, betaine and control groups, hydropic degeneration in tubular epithelial cells (tip arrow), protein casts (thick arrow) and hemorrhage (thin arrow) (A): Control group, (B): MI group, (C): Betaine receptor group 50 mg kg day, (D): Betaine receptor group 150 mg kg day (E): the group receiving betaine 250 mg kg day. (magnification: ×400)

**Figure 2:**
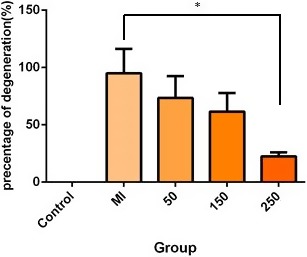
Comparison of tissue damage in terms of degeneration among the studied groups. The significance level of 0.05(*) indicates a significant difference between the group receiving betaine at a dose of 250 mg/kg compared with the MI group (*p* = 0.025)

To confirm renal damage, kidney tissue sections from all groups were analyzed by optical microscopy. Hydropic degeneration damage in tubular epithelial cells, protein casts and hemorrhage have been shown in Figure 1A–E. Then, these damages were assessed using ratio test. According to the results, compared to other treatment groups, less damage to the kidney tissue at a dose of 250 mg/kg betaine indicate the beneficial effects of betaine compared with other doses (50 and 150 mg/kg) (Figure 2).

### 3.2 Serum TNF‐α level

**Figure 3:**
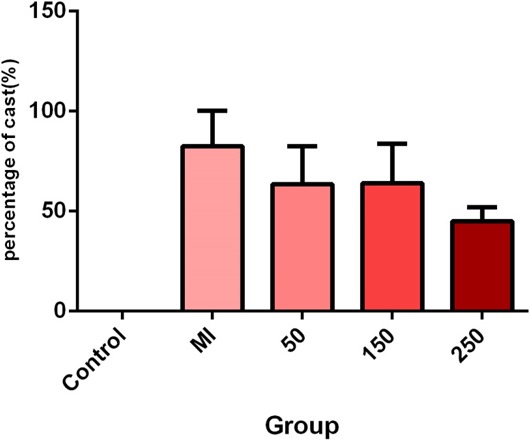
Comparison of tissue damage in terms of cast formation among the studied groups

**Figure 4:**
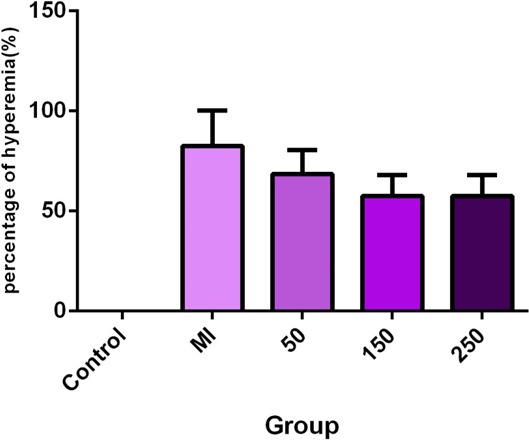
Comparison of tissue damage in terms of hyperemia among the studied groups

**Figure 5:**
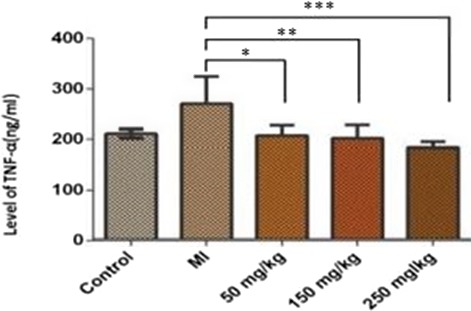
Comparison of serum TNF‐α level in the studied groups. The statistical analysis was performed by one‐way ANOVA and *p* < 0.05 was considered as statistically significant. Based on the results, only the differences between MI treatment groups and 50‐mg treatment groups, MI groups and 150‐mg treatment groups, MI and 250‐mg treatment were statistically significant (*p* = 0.0185), (*p* = 0.0093), and (*p* = 0.0010) displayed by *, ** and ***, respectively

According to the findings, since serum levels of TNF‐α were normally distributed, therefore, a one‐way ANOVA and tukey follow‐up test were utilized. The results showed that the injection of isoprenaline did not significantly affect the serum TNF‐α level between the control and MI groups; however, it increased the serum TNF‐α level. In addition, the comparison of serum TNF‐α level between the MI group and the treated groups with betaine at doses of 50, 150, and 250 mg/kg showed a significant decrease in all three treatments compared to the MI group. However, a high significant difference was found in the group treated with a daily betaine dose of 150 mg kg^−1^ day^−1^ (*p* = 0.0010) (Figures 3, 4, 5).

### 3.3 Serum creatinine level

**Figure 6:**
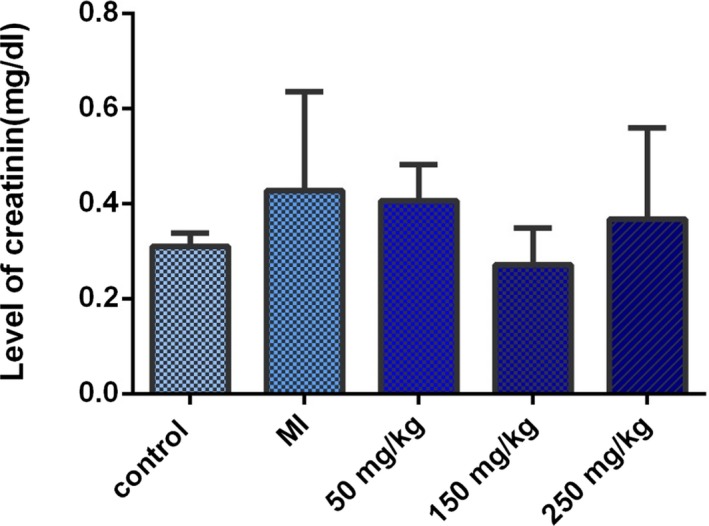
The serum creatinine level in the rats of control group, MI group and the groups treated daily with 50, 150 or 500 mg/kg betaine, and *p* < 0.05 was considered statistically significant. The highest serum creatinine level was found in the group treated daily with 50, 150, or 500 mg/kg betaine. No significant difference was observed in all groups

The comparison of serum creatinine level in the control and MI groups showed an elevated serum creatinine levels in the MI group, which was not statistically significant. The serum creatinine level was also decreased in treated groups compared with the MI group, which was higher in the group treated with betaine at a dose of 150 mg kg^−1^ day^−1^. However, no significant difference was observed in all groups (Figure 6).

### 3.4 Expression of nitric oxide synthase enzyme

**Figure 7:**
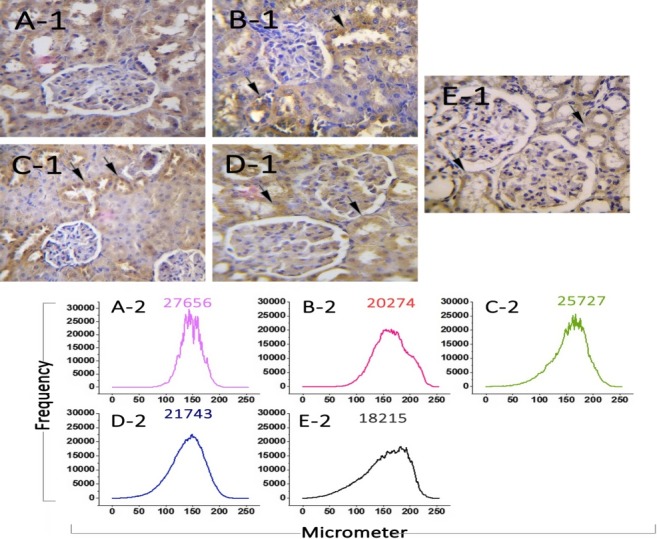
Immunohistochemical analysis of NOS expression. No significant increases were found in transverse sections of the kidney in the studied groups compared to the control group. The dispersion of positive NOS‐positive cells per µm^2^ and the coloration intensity for the chromogen in all groups were expressed based on the mean ± standard deviation. In immunohistochemical staining, brown cells display the number of cells with NOS protein. (A1, 2): Control group, (B1, 2): MI group, (C1, 2): Betaine group (50 mg kg day, (D1, 2): Betaine group (150 mg kg day) and (E1, 2) Betaine group (250 mg kg day)

**Figure 8:**
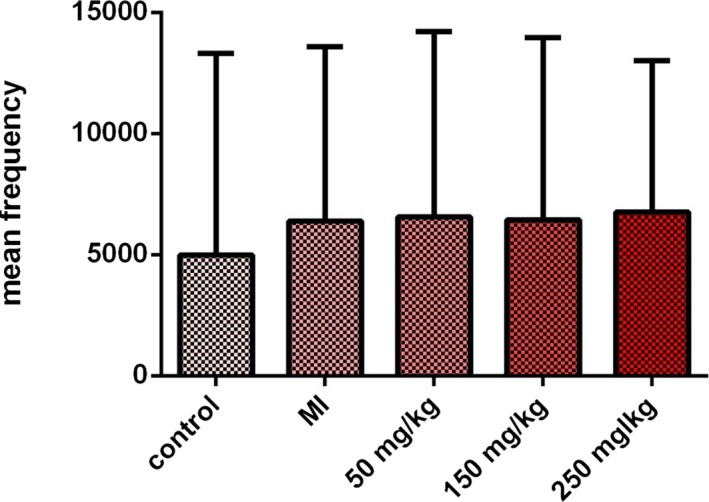
The mean NOS‐positive response in the rats of control group, MI, and the treated groups with betaine at doses of 50, 150, and 250 mg kg day^−1^. A *p* < 0.05 was considered statistically significant. The highest and lowest mean positive reaction for nitric oxide synthase was observed in treatment (50 mg) and control groups, respectively. Despite the change in the mean NOS‐positive response, no significant difference was found in all groups

The analysis of immunohistochemical sections did not show a significant difference between the different groups. The images of the slides and the corresponding diagrams have been presented in Figure 7. The mean positive NOS response in male rats is also given in Figure 8.

## 4 DISCUSSION

The present study investigated the protective effects of Betaine on kidney injury caused by induced‐myocardial infarction by isoprenaline. According to the results, the injection of isoprenaline decreased the incidence of hypertension and renal damage represented by histopathological changes and hydropic degeneration in tubular epithelial cells in the kidney tissue. Further, betaine at doses of 50, 150, and 250 mg kg day^−1^ decreased serum creatinine level in comparison with the MI group, with the highest reduction was found in betaine data dose of 150 mg kg^−1^ day^−1^ (which was not statistically significant). In the treatment group, betaine injections at doses of 50, 150, and 250 led to a decrease of TNF‐α level compared to the MI group and an elevated betaine level resulted in a significant dose‐related decrease in serum. It was also shown that the administration of betaine at doses of 50, 150, and 250 decreased the protein content of nitric oxide synthase enzyme‐2 in the rats of MI and treatment groups. Previous studies showed that an increase in serum creatinine level is associated with acute MI (Cakar, Gunduz, Vatan, Kocayigit, & Akdemir, 2012), which was congruent with our study and was not statistically significant; this could be due to different sampling time. Given the lack of statistical significance in creatinine levels in the isoprenaline group compared to the control and MI groups, this study did not detect any evidence for the role of isoprenaline in renal damage improvement; however, it was clearly evident that high doses of betaine (150 and 250 mg kg day^−1^) resulted in a decrease in serum creatinine level. An increase in the serum creatinine level by 0.5 mg suggests deteriorating kidney damage in the rats; whereas, it has been found that betaine in high doses may play a protective role. The mechanism of kidney damage can be deduced from the treatments provided. Lekston et al. identified *N*‐acetylcysteine (NAC) and osmotic pressure modulating agents such as mannitol, sodium bicarbonate, and statins as neuroprotective agents (Lekston, Kurek, & Tynior, 2009). Although this study did not examine the osmomodulatory effects of betaine, they may partly explain betaine properties, which need further investigation. The present study used betaine as a compound with protective effects. Generally, the effects of betaine can be reviewed in three domains: Antioxidant effects of betaine induced by antioxidant enzymes such as superoxide dismutase, glutathione peroxidase, which have been suggested as the first line of defense against renal injury (Alirezaei, Jelodar, Niknam, Ghayemi, & Nazifi, 2011). A number of studies have shown the central role of oxidative stress in the pathogenesis of myocardial infarction, which can lead to infarction or mediates subsequent damages (Hori & Nishida, 2008; Madamanchi, Vendrov, & Runge, 2005). Moreover, some studies have reported the protective effects of two important antioxidants of ascorbate and histidine against isoprenaline injuries, which can be attributed to decreasing free radicals level (Davel et al., 2008). It is likely that betaine prevents damage to the kidneys by decreasing cardiac damage; that is, the protective effects of betaine inhibits serious damage to the heart and consequently protects it against renal injury. The second possibility could be attributed to the direct effects of betaine on the renal system. If oxidative stress and inflammation (caused by isoprenaline and myocardial infarction due to oxidative stress) are considered as pathologic factor for renal injury, this may explain the protective role of betaine via these mechanisms. This finding corroborates the results of the study by Liu YL et al., who suggested that betaine improves kidney function in hyperemic mice (Liu et al., 2014). A review of literature supported the antioxidant effect of betaine and the reduction of kidney damage caused by cadmium. The findings of the study by Hagar et al. aimed at evaluating the effect of betaine supplementation on renal function after cadmium poisoning showed that betaine decreased the thiobarbituric acid reactive substances and resulted in an increase in antioxidant levels against cadmium (Hagar & Al Malki, 2014). Likewise, Ali Mirzai et al. confirmed these protective effects against levodopa (Alirezaei, 2015). In this study, TNF‐α factor levels were shown to increase in the group received isoprenaline. Davel et al. found that NF‐κB transcription factor is induced in the presence of isoprenaline resulting in increased inflammatory factors (Davel et al., 2008), which is in agreement with those results of our study. Decreases in the TNF‐α level can be due to the inactivation of transcription factor NF‐κB (nuclear factor kappa‐light‐chain‐enhancer of activated B cells). Cell surface receptors can activate the transcription factor (Kawai & Akira, 2007). However, in reviewing the literature, no study was found regarding the interplay of betaine with cell surface receptors in activating transcription factors. Therefore, the potential activity of the NF‐kB transcription factor, followed by TNF inflammatory factor, as well as its inflammation and damage can be reduced by inhibiting oxidative stress. This result may be explained by the fact that oxidative stress plays a crucial role in the inflammation by activating intracellular signaling pathways (Liu et al., 2016). Various studies supported the anti‐inflammatory effects of betaine (Liu et al., 2016; Rajaie & Esmaillzadeh, 2011). Kidney damage prevention seems to take place through reducing oxidative stress and inflammation. If lowered inflammation results in decreases in renal damage, then it is likely that a low level of TNF‐α protects the kidney or heart. Krown et al. (1996) found that TNF‐α induced apoptosis in cardiac cells (Krown et al., 1996). Several studies have supported the central role of nitric oxide synthase enzyme in renal damage (Misseri et al., 2005). Therefore, this combination of findings provides some support for the idea that inflammation caused by isoprenaline injection can be inhibited by betaine resulting in decreased renal damage. Several studies reported the pro‐apoptotic impacts of TNF‐α on the renal cells. It is thus likely that betaine leads to the modulation of isoprenaline‐induced inflammatory response in the renal cells. Additionally, our results did not show a significant difference between the studied groups in terms of protein content of nitric oxide synthase enzyme‐2. The nitric oxide synthase‐2 enzyme is recognized as one of the main elements of oxidative and necrotic damage in cardiovascular and renal diseases. These results suggest that no changes in the groups could be due to the effects of betaine; it is possible that betaine suppressed the potential elevation of the nitric oxide synthase enzyme. The results of the study by Wand D et al. on the rat model showed that the inhibition of enzyme activity of nitric oxide synthase 1 caused a decrease (20%) in the area involved in cardiovascular damage (Wang, Yang, Liu, Carretero, & LaPointe, 1999). This result also concords with earlier studies conducted by Feng et al. the evidence from this study suggests that mice with mutagenesis induced by the nitric oxide synthase‐1 enzyme are more resistant and have lower mortality compared to wide type in cardiac damage after myocardial infarction (Feng et al., 2001). Therefore, betaine might play its protective role through these mechanisms.

## 5 CONCLUSION

Overall, the present study investigated the tissue structure and renal function of all male rats after isoprenaline‐induced myocardial infarction and treatment with betaine. Decreased level of TNF‐α and creatinine level resulted in lowered inflammation and kidney damage. Betaine treatment in rats reduced the TNF‐α level and creatinine level (without no significant decrease), resulting in lowered inflammation and kidney damage. In addition, histologic and immunohistochemistry studies suggested the improvement of tissue damage caused by isoprenaline in the kidney. Our results also indicated that the betaine effect is dependent on the dosage and stabilizing and strong effects of betaine on the structure and function of kidney are obtained at a dose of 250 mg other than other doses (150 and 50 mg/kg).

## 6 RECOMMENDATIONS FOR FURTHER WORK

Perform studies with more statistical population. Perform molecular studies in order to investigate other inflammatory factors including the interleukin family. Perform studies aimed at assessing renal damage in terms of apoptosis and necrosis. Carrying out studies aimed at examining the effects of betaine protection through simulator.

## CONFLICT OF INTEREST

None declared.
